# Ovarian serous carcinoma: recent concepts on its origin and carcinogenesis

**DOI:** 10.1186/1756-8722-5-8

**Published:** 2012-03-09

**Authors:** Jie Li, Oluwole Fadare, Li Xiang, Beihua Kong, Wenxin Zheng

**Affiliations:** 1 Department of Obstetrics and Gynecology, Qilu Hospital, Shandong University, 107 W. Wenhua Road, Jinan, Shandong, China 250012; 2 Department of Pathology, University of Arizona College of Medicine, Tucson, AZ, USA; 3 Department of Pathology, Microbiology and Immunology, Vanderbilt University School of Medicine, Nashville, TN, USA; 4 Department of Obstetrics and Gynecology, University of Arizona, Tucson, AZ, USA; 5 Arizona Cancer Center, University of Arizona, Tucson, AZ, USA; 6 Department of Pathology, University of Arizona, 1501 N. Campbell Avenue, #5224A, Tucson, AZ 85724, USA

**Keywords:** Ovarian cancer, Fallopian tube, Carcinogenesis, Serous carcinoma, p53 signatures

## Abstract

Recent morphologic and molecular genetic studies have led to a paradigm shift in our conceptualization of the carcinogenesis and histogenesis of pelvic (non-uterine) serous carcinomas. It appears that both low-grade and high-grade pelvic serous carcinomas that have traditionally been classified as ovarian in origin, actually originate, at least in a significant subset, from the distal fallopian tube. Clonal expansions of the tubal secretory cell probably give rise to serous carcinomas, and the degree of ciliated conversion is a function of the degree to which the genetic hits deregulate normal differentiation. In this article, the authors review the evidentiary basis for aforementioned paradigm shift, as well as its potential clinical implications.

## 

Worldwide, approximately 225,500 women are diagnosed with ovarian cancer annually, with an estimated 140,200 associated deaths [1]. Although ovarian cancer accounts for only 3% of all cancers in women, it has one of the highest death-to-incidence ratios, which has been primarily attributed to the unavailability of effective screening tools, the absence of early phase symptomatology in many patients, and accordingly, its typical presentation at advanced stages when prognosis is poor [2,3].

As has become apparent in recent years, one of the greatest obstacles to the detection of early-stage ovarian cancer was our poor understanding of its histogenesis and pathogenesis. Ovarian carcinoma was traditionally thought to originate from the ovarian surface epithelium (OSE) or ovarian epithelial inclusions (OEI), and investigative efforts at early detection have accordingly been centered on the ovary for decades. However, these efforts have not been successful, as evidenced by the fact the overall survival for women with ovarian cancer has not changed in any fundamental manner over the last 50 years [4]. Several emerging lines of evidence indicate that some traditional tenets of ovarian epithelial carcinogenesis and cellular origination are fundamentally flawed.

Patients with high-grade serous carcinoma (which constitute 60-80% of ovarian epithelial carcinomas [OEC], and which represent the archetypical "ovarian cancer") most frequently present at advanced clinical stage and have a very poor overall survival [5,6]. Therefore, understanding of the nature and development of this type is extremely important for improving the survival rate of ovarian cancer patients as a group. Recently, studies of both asymptomatic women with germline *BRCA1 *or *BRCA2 *mutations as well as those from the general population with pelvic serous carcinoma, have detected precancerous or early cancerous lesions - serous tubal intraepithelial carcinoma (STIC) - in the fallopian tubal fimbria [7-9]. Furthermore, a spectrum of potential precursor lesions to serous carcinomas, including the 'p53 signatures' and the 'secretory cell outgrowth', have similarly been described in the fimbria [10,11]. From those and other studies, the fallopian tube has emerged as an important potential source for female pelvic serous carcinomas, resulting in a paradigm shift that will likely have important implications for future detection, therapy and prevention in ovarian cancer. This review will discuss the recent advances in the field of ovarian or pelvic serous histogenesis and carcinogenesis, as well as the potential clinical applications of these advances.

## Dualistic model of serous carcinoma

Based on morphologic, immunohistochemical and molecular data, a broader, dualistic model of ovarian carcinogenesis has been proposed that classifies ovarian carcinomas into 2 groups: Type I and Type II. Type I tumors include low-grade serous carcinomas (LG-SC), low-grade endometrioid carcinomas, clear cell and mucinous carcinomas, and Brenner tumors. Type I tumors are not clinically aggressive, generally present at early stage, rarely harbor *TP53 *mutations, but instead display mutations involving specific cell signaling pathways, including *KRAS, BRAF, ERBB2, PTEN, CTNNB1, PIK3CA, ARID1A, and PPP2R1A*. Type II tumors, which include high-grade serous carcinomas (HG-SC), high-grade endometrioid carcinomas, malignant mixed mesodermal tumors (carcinosarcomas), and undifferentiated carcinomas, frequently display *TP53 *mutations and are genetically unstable [12].

LG-SC is thought to develop in a stepwise fashion, sequentially from OEIs or serous cystadenoma, then to serous borderline tumor, and eventually to invasive carcinomas [13-15]. In contrast, HG-SC, the prototypic of type II ovarian cancer, is a highly aggressive neoplasm, and afflicted patients almost always present at an advanced stage. HG-SC is characterized by high levels of genetic instability, a very high frequency of *TP53 *gene mutations, and low frequency of the molecular aberrations that typify Type I carcinomas including *KRAS *and *BRAF *mutations. Therefore, this dualistic model has clinical, pathologic and molecular evidentiary support, and provides a valuable framework for studying and analyzing the larger subject of ovarian carcinomas.

## Cell of origin of serous carcinoma

The origin of ovarian cancer has perplexed investigators for decades. The more prominent theories that have been preferred are discussed below:

## The theory of OSE as origin of OEC and its drawbacks

The hypothesis that OSE is the site of origin site for OEC is based on relatively weak histological arguments, its wide acceptance notwithstanding. The OSE, which is continuous with the mesothelial lining of all pelvic organs, bears no resemblance to OECs. Some studies have showed that the OSE cell is an uncommitted mesothelial cell, which has a phenotypic plasticity, as demonstrated by its reactivity with antibodies indicative of multiple lines of differentiation [16,17]. The OSE was assumed to invaginate into the underlying stroma to form OEI [16]. These inclusion cysts, with their newly acquired müllerian phenotype (by metaplasia), could then undergo malignant transformation resulting in carcinomas corresponding to the different cell types (serous, endometrioid, clear cell, mucinous and transitional cell) [18].

The OSE theory for the genesis of OECs has a number of limitations. If one takes the point of view that carcinomas arise from, or at minimum differentiate towards, a cell type native to the organ in which the carcinomas arose, then the following three points are salient: 1) The normal ovary has no constituents that resemble the OECs, and some biomarkers such as HOXA and PAX8 are extensively expressed in OECs, but not in OSE [18-20]. 2) OSE overlying the ovaries is continuous with mesothelium lining of all pelvic and abdominal organs. The OSE or mesothelium, is embryologically distinct from Müllerian epithelia in general; 3) A true precursor lesion of OECs has rarely, if ever, been found in the ovaries; Although OEIs lined with ciliated cells are frequently observed in the ovarian cortex, well-documented examples of what can be interpreted as a transition from these cystic structures to high grade carcinoma have not been reported. These simple observations are arguments against the theory that OEC originate from the OSE with any significant frequency.

## Secondary müllerian system theory

Some tumors that are morphologically identical to ovarian carcinomas can occasionally be found outside the ovary, even though the ovaries, or indeed the ovaries, fallopian tubes and uterus, had previously been removed [21-23].

Therefore, an alternate theory was proposed by Lauchlan [24], in which all of the Müllerian-type epithelium found outside the primary Müllerian system (uterus, cervix, and fallopian tubes) were termed as "secondary Müllerian system", including OSE, OEIs, paraovarian/paratubal cysts, rete ovarii, endosalpingiosis, endometriosis and endocervicosis. Lauchlan proposed that all tumors with a Müllerian phenotype (serous, endometrioid, and clear cell) in the pelvis are derived from these müllerian-type epithelia either directly or by a metaplastic process. This theory provided a straightforward explanation for the presence of ovarian-like tumors arising outside the primary Müllerian system. Lauchlan also proposed that endosalpingiosis, endocervicosis, and endometriosis can evolve into one another through metaplasia. Although it is still unclear what conditions or factors promotes this transition from one state to the other, metaplasia from endosalpingiosis to endocervicosis or to endometriosis are commonly observed in ovarian pathology [25]. Lauchlan's visionary hypothesis remains instructional in clinical practice and ovarian cancer research. Additionally, the secondary Müllerian system succintly explains why the epithelial tumors in ovary and in pelvis are morphologically identical to those tumors within the primary Müllerian system. However, it does not specifically address whether tumors that have traditionally been classified as "ovarian" in origin may have an extraovarian origin.

## The tubal theory: the fallopian tube as the site of origin for "ovarian" high grade serous carcinoma

The fallopian tube has traditionally played a minor role in female adnexal pathology. However, the past decade has seen the emergence of a robust body of evidence, including clinical-pathological and molecular findings, that lend significant credence to the notion that "ovarian" HG-SCs result from the a clonal expansion of the secretory cells in the distal fallopian tube, rather than the ovary [9,26-29].

### Clinical-pathological evidence

Since most of ovarian carcinomas that are identified in BRCA mutation carriers are HG-SCs, examination of prophylactic salpingo-oophorectomy specimens should theoretically reveal a precursor lesion in the ovary in at least a subset of these patients. However, early serous malignancies STICs have been found to involve the fallopian tube, rather than the ovarian surface or within ovarian cortical inclusion cysts. Piek et al were the first to describe "dysplastic changes or tubal dysplasia", which was later described as tubal lesions in transition (TLIT) [11,30] in the fallopian tubes in this setting. The authors described lesions that closely resemble HG-SCs in the fallopian tubes of 50% of a cohort of women with familial high risk for ovarian cancer undergoing prophylactic salpingo-oophorectomies [31]. There have now been numerous subsequent studies, in which fallopian tubes were more carefully examined, that confirmed the existence and incidence of STICs and early invasive tubal carcinomas in BRCA mutation carriers that underwent risk reducing salpingo-oophorectomy procedures [7-9,32-38]. Based on these studies, early serous carcinomas in these specimens occur in between 2% and 17% of cases and involve that fallopian tube mucosa up to 100%.

The next important step linking STICs with serous ovarian carcinoma was the observation that 35-70% of sporadic ovarian and peritoneal HG-SCs showed mucosal tubal involvement, including STICs. Kindelberger et al extensively examined the fallopian tubes from 55 consecutive cases of serous carcinoma [28]. They showed that over 70% of serous carcinomas involved the endosalpinx and approximately half contained STICs. To further confirm the shared origin of these ovarian serous carcinomas with their coexisting STICs, they also analyzed *TP53 *mutations in both sites from five cases. Identical mutations were detected in both sites for all cases. Salvador et al studied 12 cases of high-grade serous carcinoma, of which 10 cases showed either unilateral tubal mucosal involvement by STICs (7/12, 58.3%) or tubal obliteration ipsilateral to the dominant ovarian mass (3/12, 25%) [39]. Further analysis of chromosomal copy number changes by FISH demonstrated similar results in 3 of 5 cases comparing ovarian serous tumors with synchronous fallopian tube serous carcinoma, providing some additional support for a common, monoclonal origin. Another study from the same group examined 45 cases of primary peritoneal serous carcinoma and found that 9 out of 26 cases (34.6%) with incomplete tubal sampling and 9 out of 19 cases (47.4%) that underwent complete examination of the tube had STIC [29]. The above observations and similar other studies, gave support to the proposal that STICs, which almost always were detected in the fimbria, may be the source of HG-SCs in both BRCA mutation carriers as well as women who do not have a known genetic predisposition for ovarian cancer. Finally, the spectrum of putative and possibly non-obligate precursor lesions that have been described in the fallopian tube lends additional support to the concept that the serous neoplastic process may well begin in the fallopian tube rather than ovary.

There are some potential limitations to the tubal theory. First, a significant subset of HG-SCs is not associated with STIC even after extensive sectioning of the tube [40]. Second, HG-SCs occasionally arise from serous borderline tumors and LG-SCs [41,42]. Third, the fimbria of the fallopian tube is normally in close contact with the ovarian surface at the time of ovulation. It is conceivable that when OSE is disrupted at the time of ovulation, normal tubal epithelial cells from the fimbria may be dislodged and implant in the ovary to form OEIs (also called endosalpingiosis) [43]. Therefore, some HG-SCs may develop from OEIs [44-46]. However, although a proportion of adnexal HG-SCs may not involve the fallopian tube, this does not necessarily rule out a fallopian tube origin for these cases. An alternative explanation for STICs that are seen in the non-BRCA setting is that they represent random growth on the tubal surface from an adjacent ovarian HG-SC. Again, this possibility is mitigated by the fact that STIC is well-established in the tube, but none have been described in the ovary. Thus, the preponderance of available evidence supports the tubal theory that a significant subset, and possibly all HG-SCs originate from the fallopian tube.

### Cellular evidence

The normal epithelium of the fallopian tubes is comprised of two cell types: ciliated and secretory. STICs and their malignant invasive counterpart in the fallopian tube, as well as HG-SCs, are all comprised of only secretory cells, while the normal fallopian tube epithelium is comprised of an admixture of ciliated and secretory cells. Initial benign appearing secretory cell proliferations can be frequently identified in the fallopian tubes with *BRCA *mutations or serous carcinomas, either associated with p53 alterations (so-called p53 signatures), or found with other genetic alterations in the absence of alterations in p53 (secretory cell outgrowths or SCOUTs).

#### p53 signatures

As defined by the Crum group, a p53 signature is a segment of benign appearing epithelium showing p53 immunoreactivity in at least 12 secretory cells. These secretory cells can be contiguous or interrupted by some remaining ciliated cells, but the lesion should have a low proliferative index, as measured by MIB-1 staining (< 20%). This lesion is suggestive of early clonal expansion but falls short of a *bona fide *neoplastic proliferation.

In a study by Lee et al, p53 signatures were observed in 37% and 33% of women with and without BRCA mutations respectively. p53 signature (53%) was more frequently identified in fallopian tubes harboring STICs. p53 signature shares several attributes with STIC, including: 1) fimbrial location in > 80%; 2) intense p53 immunoreactivity; 3) involvement of the secretory cell; 4) evidence of DNA damage as manifested by punctuate immunopositivity for γ-H2AX; 5) *TP53 *mutations in approximately 60%; 6) identical *TP53 *mutations with concurrent STIC lesions; 7) occasionally, direct continuity with STIC. The presence of p53 signature in the fimbria of normal women provides evidence that suggests that secretory cells can experience sufficient toxic damage to trigger a DNA damage response under normal physiologic conditions [10]. To determine whether the same lesions also occur in the ovarian epithelium or cortical inclusions, Folkins et al examined the ovaries of 75 *BRCA *mutation carriers, only one OSE showed p53 signature and none in cortical inclusions, confirming that p53 signatures preferentially arise in fallopian tube epithelium rather than OSE [47]. Based on these findings, a sequence of pathogenetic events has been proposed that under some genotoxic events, secretory cells are prone to DNA damage, followed by *TP53 *mutation and progressive loss of cell cycle control, which eventuate in the development of carcinoma.

The frequency of p53 signatures in women with and without *BRCA *mutations is nearly the same, suggesting that p53 signature is not directly linked to this genetic risk factor [10]. It is presumed that p53 signatures rarely undergoes transition from benign to malignancy in women who do not have a *BRCA *mutation, consistent with its role as a latent precursor, which, by definition, requires an additional event to undergo this transition. In contrast, it is hypothesized that for the women who have *BRCA *mutations, they are particularly susceptible to subsequent events, such as loss of *BRCA1 *or *BRCA2 *function since they have had a preexisting germline mutation.

#### Secretory cell outgrowth

More recently, the spectrum of potential precursors in the fallopian tube was expanded to include more secretory cell proliferations, which was defined as secretory cell outgrowth (SCOUT) [48,49]. SCOUT is defined as a discrete expansion of at least 30 epithelial cells of secretory type (BCL2 expressing, p73 non-existing) that are distinct against a heterogeneous background of tubal secretory and ciliated cells [49]. A p53 signature is essentially a p53-positive SCOUT with different size requirements. SCOUTs have other characteristics that distinguish them from p53 signatures, including 1) significantly wider and more even distribution between fimbrial and proximal areas of the fallopian tube; 2) loss or reduction of PAX2 expression (so-called PAX2-null SCOUTs); 3) Lack of alterations in *TP53 *or diffuse DNA damage [49]. Some studies investigated the frequency of SCOUTs using the patterns of BCL2/p73 immunostaining in tubes from women with serous carcinoma, inherited mutations in *BRCA1 *or *BRCA2 *and benign controls [49]. SCOUTs were found to be significantly more prevalent in the serous carcinoma group and more aging patients as compared to the others [48,49]. Abnormalities in both p53 and PAX2 expression occurred in the distal fallopian tube and characterized p53 signatures, STICs and advanced pelvic serous carcinomas. All three entities can, on occasion, be demonstrated in continuity, and may share identical *TP53 *mutations and loss of PAX2 staining [49]. These findings indicate that PAX2 dysfunction is involved in HG-SC development. However, only a few SCOUTs with both abnormal p53 and PAX2 expression (p53 signatures) are linked firmly enough to pelvic serous cancers to be confirmatory of a causal relationship. Thus, future research will be required to establish whether SCOUTs are reactive or precancerous.

### Molecular evidence

The *TP53 *gene is mutated in almost 100% of HG-SC [50,51]. This mutation is not only nearly ubiquitous in HG-SC, but is also an early event in HG-SC carcinogenesis. Intense p53 immunoreactivity and *TP53 *gene mutations have been observed in STICs, and half of p53 signatures display *TP53 *mutations [10,28,29,47]. In some cases p53 signatures and STICs share identical *TP53 *mutations with the concomitant HG-SC, suggesting a clonal relationship between them [10,28]. Further evidence implicating the fallopian tube as the site of origin comes from a gene expression profiling studies, showing that the gene expression profile of HG-SC is more closely related to the fallopian tube than to the OSE [52,53]. Based on the above evidence, a plausible serous carcinogenic model has emerged following a spectrum of visible epithelial alterations with the following stages: p53 signature, tubal lesions in transition (tubal dysplasia), STIC, and the full blown serous carcinoma.

## The tubal theory: the fallopian tube as the site of origin for "ovarian" low grade serous carcinoma

LG-SC is thought to evolve in a stepwise fashion from OEIs/serous cystadenomas to serous borderline tumors to invasive carcinoma [54,55]. Our group recently showed that the majority of OEIs are derived from the fallopian tube rather than OSE, and that the tubal secretory cell is the likely the cell origin of LG-SC [56]. This central role of the fallopian tube in various components of this sequence has received further evidentiary support from other groups [57,58].

### Clinical-pathological evidence

Development of LG-SC in a step-wise fashion from OEIs is supported by the following morphologic observations. First, the majority of serous cystadenomas are thought to be derived from OEIs, as both display a morphologically and immunophenotypically similar epithelial lining, and the diagnostic criterion separating OEIs from serous cystadenoma is arbitrarily made at the 1 cm size threshold [59]. Second, the majority of LG-SCs are associated with borderline tumors [15] and histological transitions from serous cystadenomas to borderline tumors are observed in nearly 75% of cases [60]. Third, foci of true early invasion in serous borderline tumors resemble LG-SC [60-63]. Additionally, several studies have shown that micropapillary serous borderline tumors have a higher frequency of invasive implants as compared with typical serous borderline tumors, and those implants are histologically identical to LG-SC [64,65]. All these morphologic observations support a model wherein LG-SC evolves from OEIs/serous cystadenomas via a serous borderline tumor.

### Cellular evidence

Since OEI may represent an initial lesion in the process of LG-SC development, demonstration of OEI origination may provide insights into the origin of LG-SC. Recently, our group evaluated the morphologic and immunophenotypic features of OEIs, OSE, serous tumors (cystadenomas, borderline tumors, LG-SCs), and distal tubal epithelium to gain a significant insight into the origin of LG-SCs [56]. In our study, we found there were two types of OSE. The vast majority OSE displayed a mesothelial phenotype (calretinin+/PAX8-/tubulin-) and a low proliferative index (0, 12 per 1000), while about 4% of cases displayed foci with tubal phenotype (calretinin-/PAX8+/tubulin+). Although the epithelium with tubal phenotype was only found in 4% cases, it did show that benign tubal epithelia are able to implant on the ovarian surface and architecturally simulate 'OSE' microscopically. In contrast, there were also two types of OEIs, while their proportional distributions were significantly different from those of OSE. Most (78%) of the OEIs displayed a tubal phenotype and had a significantly higher proliferative index than OSE, indicating that in most cases, OSE and OEIs are of different cellular lineages. The fact that we found more tubal-like epithelium in OEIs than in OSE is a strong argument in support of the notion that most OEIs are not derived from the OSE. The most straightforward explanation is the fallopian-derived OEIs represent intraovarian endosalpingiosis, which is well in line with the ideas expressed by Dubeau and Crum [30,66]. Furthermore, if the fallopian tube-derived OEIs (78%) were truly originating from mesothelium-derived OEIs through a müllerian metaplasia, the metaplastic process must be a common event and hybrid type of OEIs should be commonly found in the ovary. However, the fact that the hybrid or intermediate type of OEIs with both mesothelial and tubal phenotypes were rarely found makes another argument that mesothelium-derived OEIs undergoing metaplasia to fallopian tube-like OEIs is unlikely. In addition, mesothelium-derived OEIs seem not able to grow into a tumor mass as all these OEIs have an extremely low cellular proliferative index (similar to OSE), while fallopian-derived OEIs showed comparable proliferative activity and immuophenotypes that are similar or identical to ovarian serous tumors. From these findings, we believe that the tubal derived OEIs are likely originated from tubal epithelia, and are the likely precursors of serous cystadenomas, borderline tumors and LG-SCs, respectively.

We also studied the proportional distribution of ciliated cells from OEI/cystadenomas to borderline tumors to LG-SCs, and found that there was a progressive decrease in the population of ciliated cells as evidenced by increasing secretory/ciliated cell ratio. This suggested that LG-SCs are consequences of a clonal expansion of tubal secretory cells, as is likely in high-grade serous carcinogenesis.

Other groups also linked the LG-SC to the fallopian tube in their recent studies. Kurman et al recently identified a fallopian tube lesion, designated as "papillary tubal hyperplasia" (PTH) and defined as "tubal proliferations that exhibited papillary tufting and detached clusters of bland epithelium frequently, but not always, associated with psammona bodies". The clusters of "epithelial cells and small papillae were found floating in the lumen or protruding from the tubal mucosa into the lumen" [57]. On the basis of the findings in their study, they proposed a model for the origin and development of the spectrum of pelvic low-grade serous proliferations. The process begins with chronic inflammation, leading to tubal hyperplasia, which, if it progresses to PTH, can shed and implant tubal epithelium on ovarian and peritoneal surfaces, resulting in a variety of low-grade serous proliferations including serous borderline tumors, noninvasive epithelial implants, and endosalpingiosis. Another study by Laury et al showed that PAX2-null SCOUTs were more frequently found in the fallopian tube of women with serous borderline tumors and the loss of PAX2 expression occurred in most serous borderline tumors [58]. In particular, they identified two cases with discrete multifocal papillary SCOUTs in the fallopian tubes, which they thought were associated with serous borderline tumors. This lesion is similar to the PTH described by Kurman's group. However, we feel it is too premature to suggest PTH as the true precursor for LG-SC. First, OEIs were widely accepted as the origin of LG-SC, which usually presented with a mixed of ciliated and secretory cell rather than pure secretory cell proliferation [56]. Second, this kind of papillary proliferation is rarely found in the fallopian tube in our practice, which is disproportional to the frequency of LG-SCs. Third, if PTH implanting on the ovarian surface is common, the subsequent LG-SC should be more commonly presenting as exophytic tumors rather than endophytic. In reality, it is rarely to see exophytic LG-SC in pathology practice. Therefore, based on our understanding, it is unlikely that PTH serves as the main precursor source for LG-SC.

## Carcinogenesis of ovarian serous carcinoma

### Molecular carcinogenesis of HG-SC

Recently published findings from the Cancer Genome Atlas (TCGA) project confirmed previously reported findings and provided significant new insights into the molecular events that underlie HG-SC [67]. In that study, the authors analyzed mRNA expression, miRNA expression, promoter methylation, and DNA copy number in 489 HG-SCs, as well as the coding genes in 316 tumors. HG-SC displays a remarkably high degree of genomic disarray, with 113 significant focal DNA copy number aberrations, and promoter methylation events involving 168 genes. The principal molecular events involving HG-SCs involve the *TP53*, *BRCA1 *and *BRCA2 *genes. Mutations in *TP53 *were seen in at least 96% of cases, and *BRCA1/2 *alterations were seen in 22% of tumors due to a combination of germline and somatic mutations. Six other statistically recurrently mutated genes were identified, including *RB1, NF1, FAT3, CSMD3, GABRA6*, and *CDK12*, but only in less than 6% of cases.

In general, most of the mutations in *TP53 *are frameshift or nonsense mutations that increase the stability of the altered and truncated protein, leading to accumulation detectable by immunohistochemistry. However, the mutations are sometimes insertions, deletions, or stop codons leading to lack of p53 production. Intense p53 staining and *TP53 *mutations have been observed in p53 signatures and STIC of fallopian tubes [10,28,29,47,68]. Thus, to date, *TP53 *is the earliest change that has been associated with HG-SC precursor lesions, and appears to be an obligatory, as well as early event for HG-SC. Interestingly, an increased risk of ovarian cancer is not associated with Li-Fraumeni syndrome (where *TP53 *mutations are characteristic), especially considering that p53 signatures foci in distal fallopian tube are much more numerous in patients with Li-Fraumeni syndrome [69]. In contrast, there is little, if any, difference in the frequency of p53 signatures in *BRCA *mutation carriers versus non-carriers [10], which suggest that although *TP53 *mutations are necessary in the genesis of HG-SC, they are not sufficient to trigger a sequence of neoplasia.

The *BRCA *genes encode nuclear proteins that participate in DNA repair via non-error prone homologous recombination [70]. *BRCA1 *or *BRCA2 *gene mutations represent a high-risk factor for HG-SC, and women harboring such mutations have a 30% to 70% chance of developing ovarian cancer by the age of 70 [71]. This mutation usually occurs in hereditary HG-SC cases and the frequency of germline *BRCA1 *or *BRCA2 *mutation is as high as 18% in population-based cohorts of women with serous carcinoma. As previously noted, up to 22% of HG-SC have germline or somatic mutations in *BRCA1/2*. Approximately 11% of HG-SCs have lost *BRCA1 *expression through DNA hypermethylation, and that epigenetic silencing of *BRCA1 *is mutually exclusive of either *BRCA1 *or *BRCA2 *mutations [67]. Only one-wild allele of *BRCA1 *or *BRCA2 *is sufficient for DNA repair mechanism, and additional somatic loss of the wild-type allele is needed in the development of carcinoma in women with germline *BRCA *mutations. Many studies have found such LOH of *BRCA *in early ovarian serous carcinoma, STIC, and even in some OEIs/OSE in ovaries from prophylactic oophorectomy specimens, indicating that loss of *BRCA *function is an early event in HG-SCs [72-74].

*TP53 *mutations and *BRCA1/BRCA2 *inactivations are both early events in HG-SCs, and the specific carcinogenetic sequence of these gene alterations is becoming clearer. p53 signature is common in women with and without inherited mutations in *BRCA1 *or *BRCA2 *[10,47]. On the other hand, in a small series of STIC and p53 signatures analyzed in women with germline *BRCA1 *mutations, loss of the wide-type *BRCA1 *allele was observed in STIC but not in the p53 signature foci [74]. These findings indicate that loss of *BRCA1 *and/or *BRCA2 *function is not necessary for p53 signature's formation and is presumed a later event than *TP53 *mutation. Knudson's classic 2-hit hypothesis appears to be operational. For example, despite the abundance of *TP53 *mutations in LFS, inactivation of a second critical gene- such as *BRCA*- is not more likely to occur in this syndrome, leading to a similar frequency of ovarian cancer therein with that of the general population [69].

**Figure 1:**
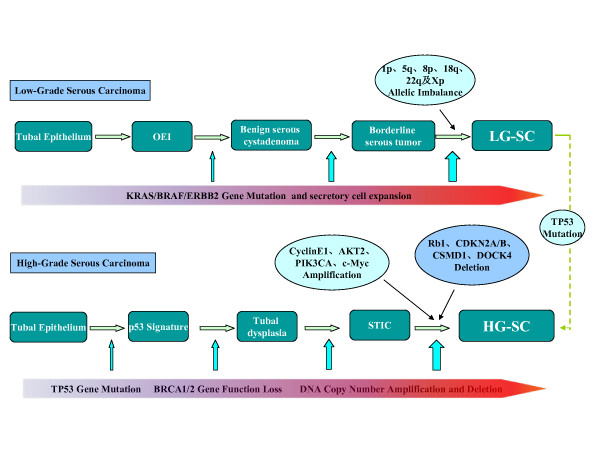
**Molecular changes of serous carcinogenesis**. Ovarian low-grade serous carcinoma (LG-SC) development starts from tubal epithelia, which invaginate into ovarian stroma to form ovarian epithelial inclusions (OEI). Further growth of OEI forms serous cystadenoma, serous borderline tumor, and LG-SC in a step-wise fashion. The most common molecular changes including KRAS, BRAF, or ERBB2 mutations are increased in this process as indicated in the upper panel. Chromosomal changes are more common prior to the development of LG-SC. In contrast to LG-SC, high-grade serous carcinoma (HG-SC) develops in a different pathway. It starts from tubal epithelia, then develop into latent precancer (p53 signature), precancer (tubal dysplasia), early cancer (serous intraepithelial carcinoma, STIC), and to full blown HG-SC. Within this process, the earliest molecular change is p53 gene mutation as indicated in the low panel. Other molecular changes are included in the figure, too. There are about 10% or less LG-SCs which can develop into HG-SC upon acquiring p53 mutation.

Finally, all HG-SCs, including the very earliest tumors show high levels of allelic imbalance [75]. Genome-wide analysis of DNA copy number alterations has demonstrated significant numbers of amplifications and deletions, among which the alterations in cyclin E1, AKT2, Notch3, PIK3CA, c-Myc, RB1, CDKN2A/B, CDK12, CSMD1, CSMD3, DOCK4, NF1, FAT3, GABRA6 are most common [67,76,77]. The main molecular changes are summarized in Figure 1.

### Molecular carcinogenesis of LG-SC

Genetically, LG-SCs are relatively stable and typically display a variety of mutations related to specific signaling pathways, including *KRAS or BRAF *but very rarely *TP53*. One study found *KRAS *mutations at codons 12 and 13 in 35% of LG-SCs and in 33% of borderline tumors [78]. Similarly, *BRAF *mutations at codon 599 were seen in 30% of LG-SC and 28% of serous borderline tumors [78]. In addition, a 12-base-pair insertion mutation of ERBB2 (encoding HER2/neu), which activates an upstream regulator of *KRAS*, has been detected in 9% of these tumors [79]. It is of interest that mutations of these three genes are mutually exclusive [80,81]. Thus, more than 60% of LG-SCs and borderline tumors have mutations of *KRAS, BRAF *or *ERBB2*. Furthermore, *KRAS *or *BRAF *mutations can be detected even in benign cystadenomas, indicating they are early events in the carcinogenesis of LG-SC [13]. Constitutive MAPK signaling will in turn lead to uncontrolled proliferation. Global gene profiling analysis showed that LG-SC had several allelic imbalances on multiple chromosomal arms, such as chromosomes 1p, 5q, 8p, 18q, 22q, and Xp, and gradually increasing chromosomal instability often presents from serous borderline tumor to borderline tumor with micropapillary structures to LG-SC [75,82], although not at comparable levels to HG-SC (Figure 1).

### Carcinogenetic model of ovarian serous carcinoma

**Figure 2:**
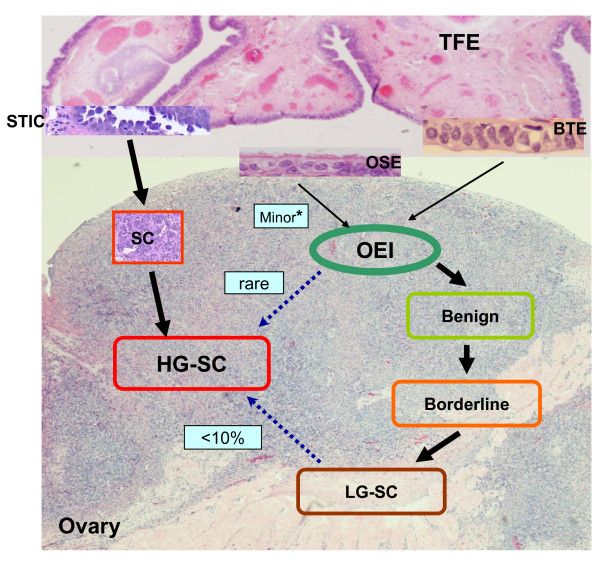
**"Ovarian" serous carcinogenesis**. The most common site of the fallopian tube for serous cancer development is the tubal fimbriated end (TFE). Benign tubal epithelia (BTE) are able to form ovarian epithelial inclusions (OEI) through an unclear pathway, then follow low-grade serous carcinoma (LG-SC) development pathway. Within this pathway, there are small amount of OEIs which are derived from ovarian surface epithelia (OSE). It is currently believed that these OSE derived OEIs are barely able to form LG-SCs. In contrast to LG-SC, HG-SC mainly derives from serous tubal intraepithelial carcinoma (STIC), which is able to stick on ovarian surface or get into ovarian stroma and expand to form full blown cancer. There are about 10% or less LG-SCs which can develop into HG-SC upon acquiring p53 mutation.

The following model for female adnexal serous carcinogenesis is supported by the currently available evidence: Both low- and high-grade serous carcinomas likely originate from the secretory cells of the fallopian tube. These secretory cell proliferations probably give rise to different types of ovarian serous carcinomas along different molecular pathways. First, under some exogenous and endogenous toxic factors, secretory cells may start to develop DNA damage. Accumulations of DNA damage will lead to chromosomal instability and a series of genetic alterations. *TP53 *mutation is an early event, which may result in clonal alterations that is the latent precursor, p53 signature. Only a subset of p53 signatures undergoes subsequent molecular events such as loss of *BRCA *function to transform to the malignant lesion STIC. Additional DNA copy number changes also play an important role in the development of malignancy. These STIC lesions form papillary tufts and the constituent cells are loosely cohesive, are easy to shed and may implant on the surface of the ovary and the peritoneum in the absence of invasive growth in the fallopian tube. This is the main carcinogenetic pathway of HG-SC. Second, the normal fallopian tubal epithelium, mostly from fimbriated end, can easily implant on the ovarian surface. Two possibilities exist for how this detachment and implantation occurs: 1) Given the close spatial relationship between the ovarian surface and the tubal fimbriated end, ovulation or non-ovulation induced disruption of the ovarian surface, may offer an opportunity for the adjacent tubal epithelium to detach and implant in the ovarian stroma [43] and 2) Adhesion of tubal epithelium on the ovarian surface, from inflammation or other factors, and ongoing stromal growth around it may eventuate in fallopian tube derived-OEI formation. The acquisition of *KRAS *or *BRAF *and possibly other mutations in tubal derived OEIs and serous cystadenomas result in their transformation to serous borderline tumors and ultimately, LG-SC [78,80,83-85]. Third, a small proportion of HG-SC may develop from LG-SC after the acquisition of additional mutations such as *TP53 *[43]. Fourth, occasionally some tube-derived-OEIs acquire *TP53 *gene mutations and develop to HG-SCs, which has been seen in women with *BRCA *mutations [46]. The latter two pathways may explain why in some HG-SCs there is neither evidence of tubal involvement nor STIC lesions. The overall ovarian serous carcinogenetic model is illustrated in Figure 2.

## Clinical implications

Previous attempts at screening for ovarian cancer, in the hope of, at minimum, intercepting the development of the disease at an early stage, have largely been unsuccessful. In one large multi-institutional prospective study of 35000 women screened with CA-125 and transvaginal ultrasounds, 70% of the women presented with advanced stage disease, which was no different from unscreened populations [86]. This suggests that cancer prevention should probably play a larger role if there is to be success in reducing ovarian-cancer related mortality.

If the fallopian tube is considered the site of origin for most adnexal serous carcinomas, then preventive strategies rather than therapeutics including immunotherapy [87], such as salpingectomies, become much more realistic. Sparing the fallopian tubes during hysterectomy for benign uterine indications provides no known physiological benefits, as the remaining tubes are completely devoid of any function in the aftermath of such a procedure, and the patient hormone profile is not altered by salpingectomy, even several months after surgery [88]. Thus, simultaneous risk-reducing salpingectomies may become more widespread whenever a hysterectomy needs to be performed for benign indications. If adnexal serous carcinomas are unequivocally shown to develop almost exclusively in the fimbria, salpingectomy alone would be sufficient to reduce the risk of adnexal serous cancer while preserving ovarian function. The resolution of these issues will likely need long term prospective trials comparing the prevalence of adnexal serous cancers between women who underwent total salpingecomy procedures and women who did not.

## Conclusion

Recent morphologic and molecular genetic studies have led to a paradigm shift in our conceptualization of the carcinogenesis and histogenesis of pelvic (non-uterine) serous carcinomas. It appears that both LG-SC and HG-SC originate from the fallopian tube. Clonal expansions of the tubal secretory cell probably give rise to both low- and high-grade serous carcinomas, and the degree of ciliated conversion is a function of the degree to which the genetic hits deregulate normal differentiation. This new paradigm has profound clinical implications in ovarian cancer prevention strategies.

## Competing interests

The authors declare that they have no competing interests.

## Authors' contributions

All authors participated in concept design, data collection and analysis, drafting and critically revising the manuscript. All authors read and approve the final manuscript.

## Authors' information

Dr. Wenxin Zheng, tenure professor of pathology and obstetrics and gynecology at the University of Arizona, is an internationally recognized gynecologic pathologist. He and his colleagues have made many original contributions in gynecologic cancers including fallopian tube is likely the source for ovarian low-grade serous carcinoma.
